# Supplementary material for: Sessile Lifestyle Offers Protection against Copper Stress in Saccharolobus solfataricus
Source: Microorganisms. 2023 May 27;11(6):1421. doi: 10.3390/microorganisms11061421 (PMC10302679; doi:10.3390/microorganisms11061421)
Supplement: Supplementary file 1 [file microorganisms-11-01421-s001.zip › microorganisms-2399198-supplementary.pdf]

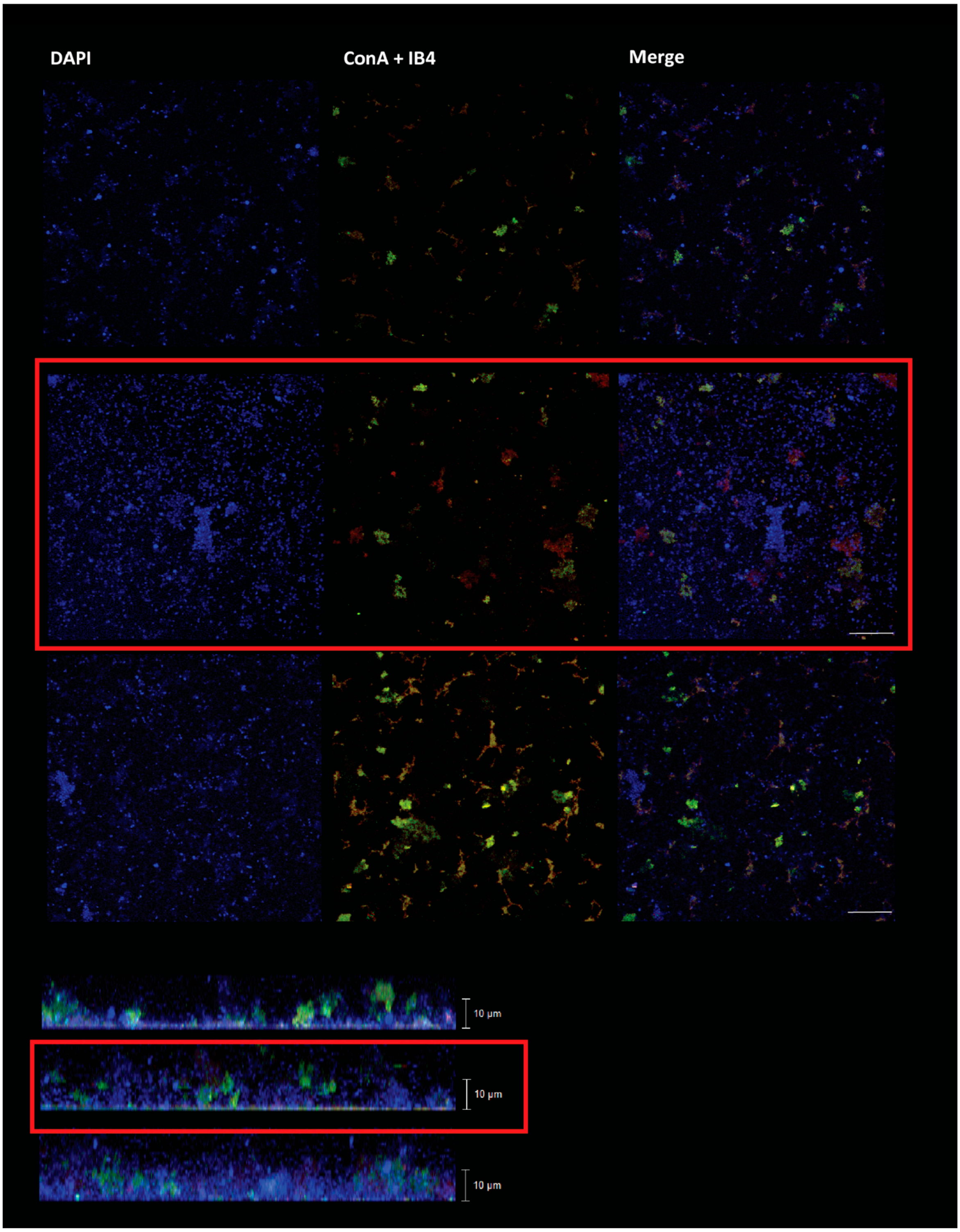

Figure S1. Replicates from biofilm CLM of *S. solfataricus*, control without Cu. The images used for the manuscript figures are indicated in red rectangles..

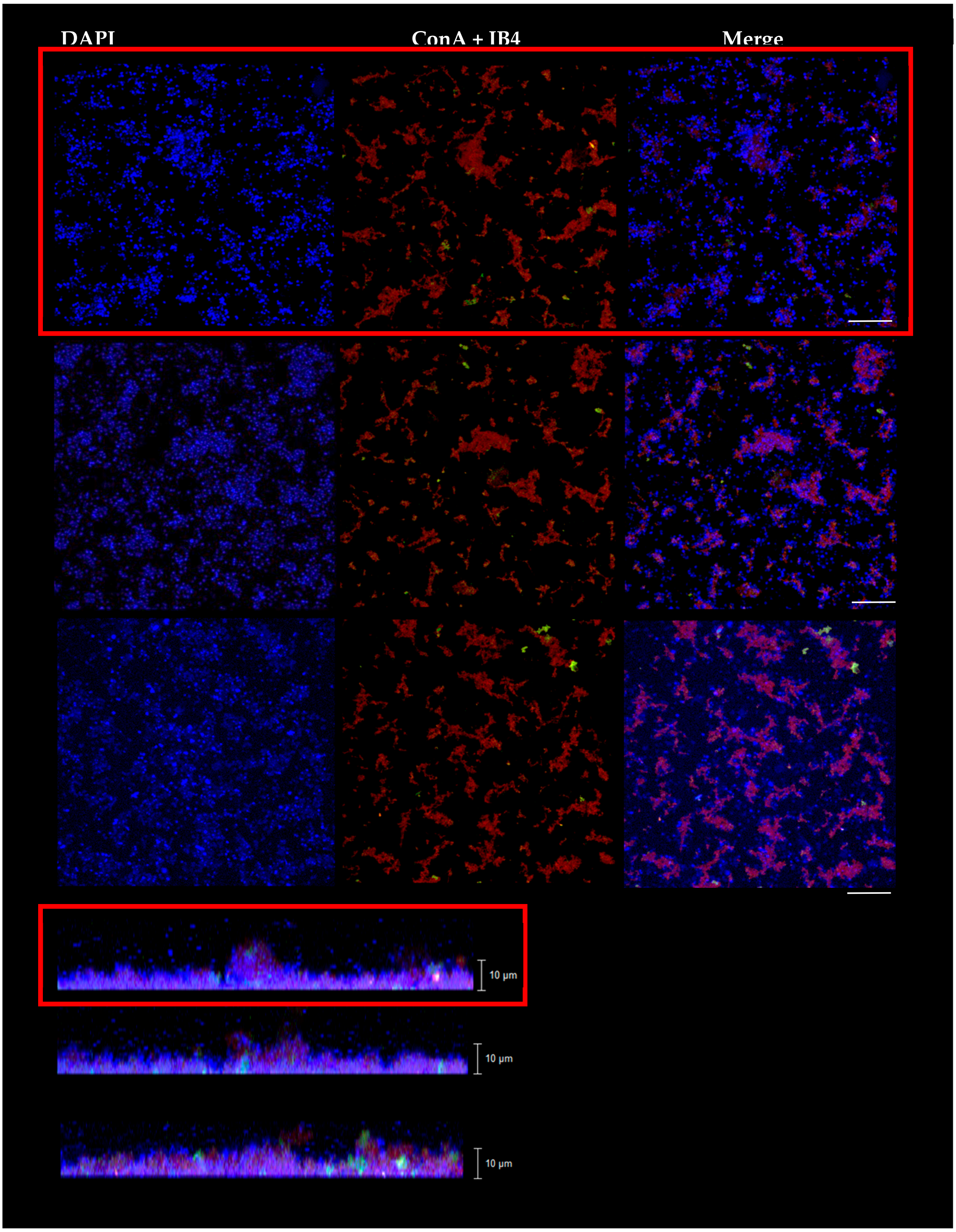

Figure S2. Replicates from biofilm CLM of *S. solfataricus* at 0.5 mM Cu. The images used for the manuscript figures are indicated in red rectangles.

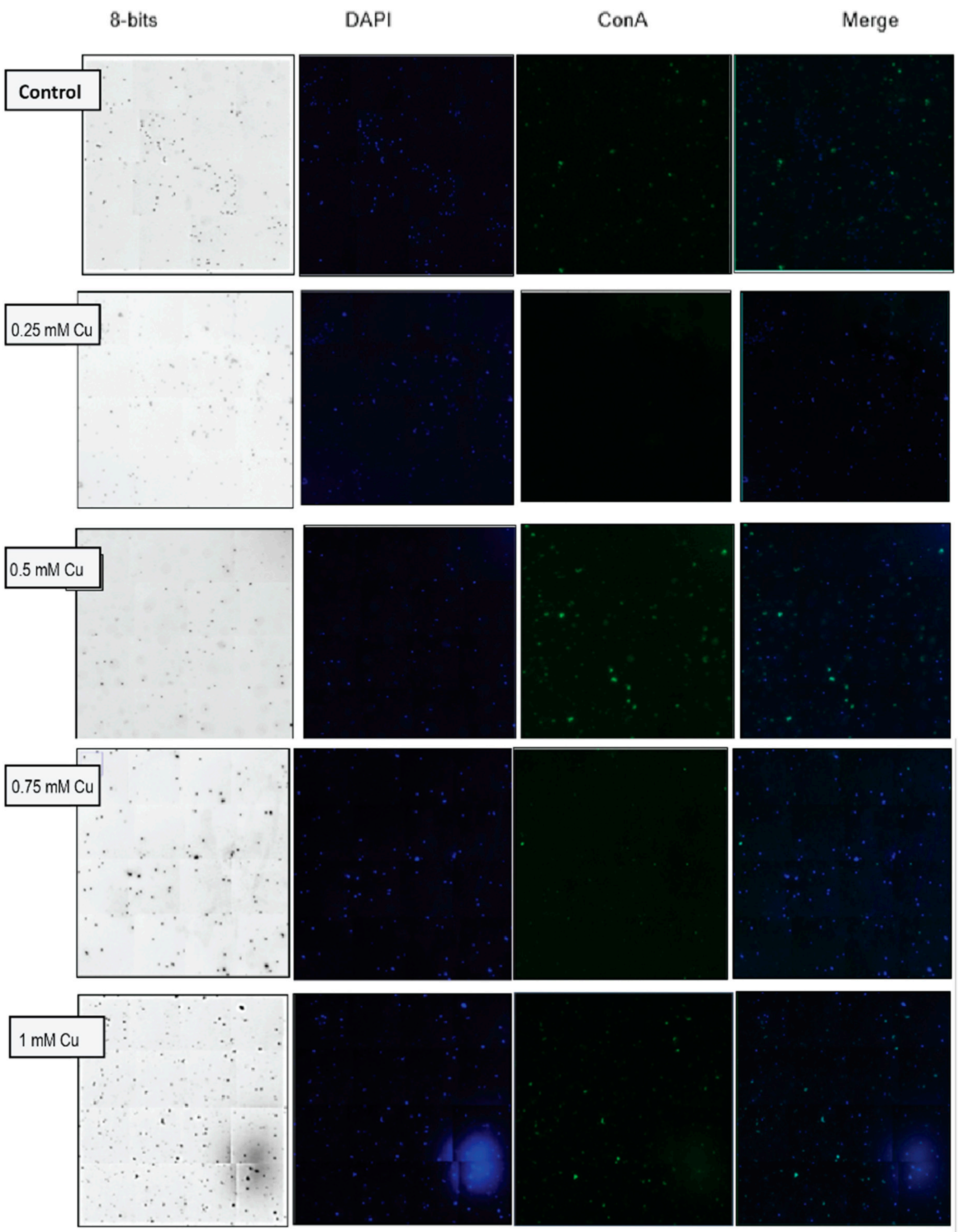

Figure S3. Illustrative images of adherence assays at the pointed Cu concentrations.

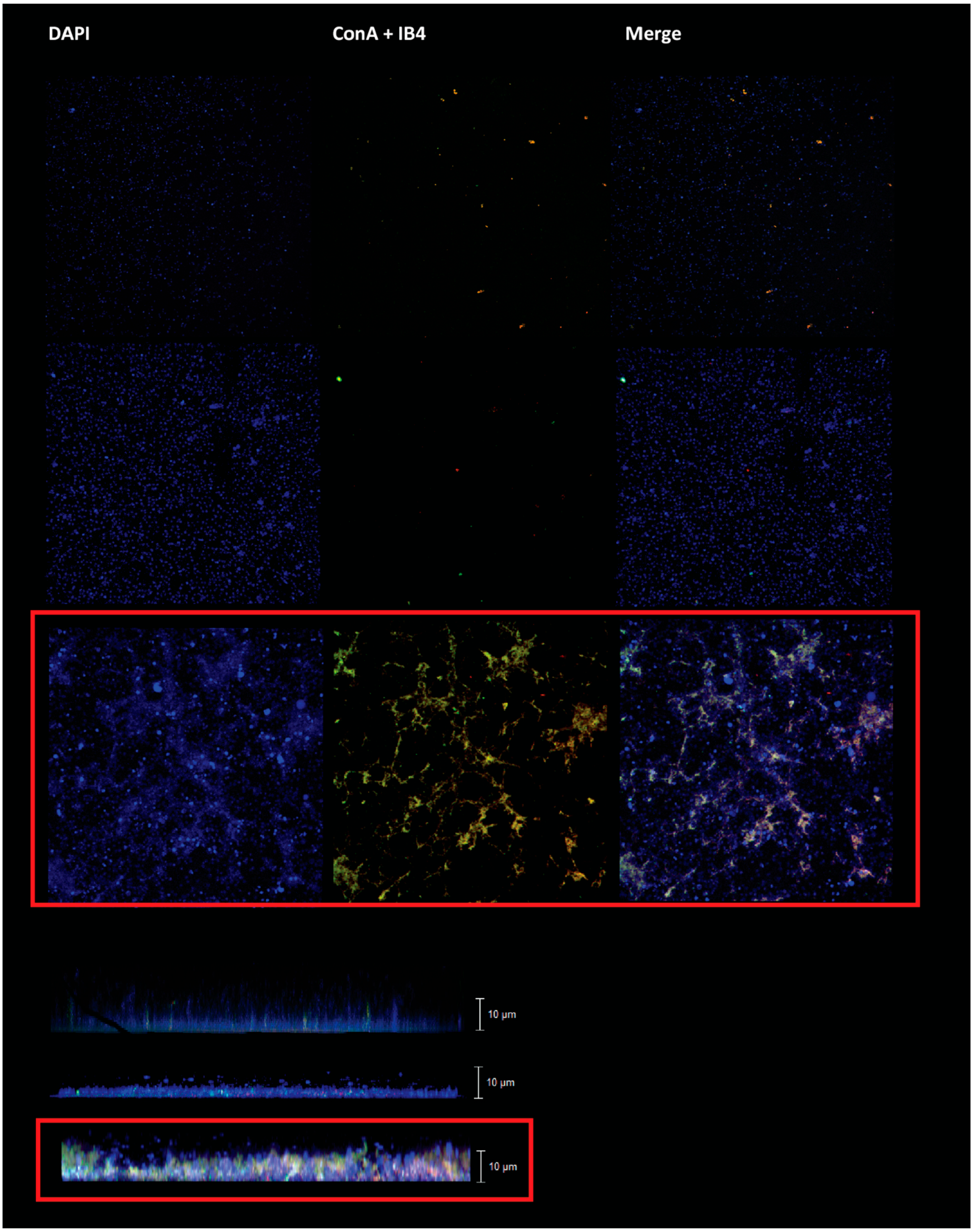

Figure S4. Replicates from biofilm CLM of *S. solfataricus* PolyP (-), control without Cu. The images used for the manuscript figures are indicated in red rectangles.

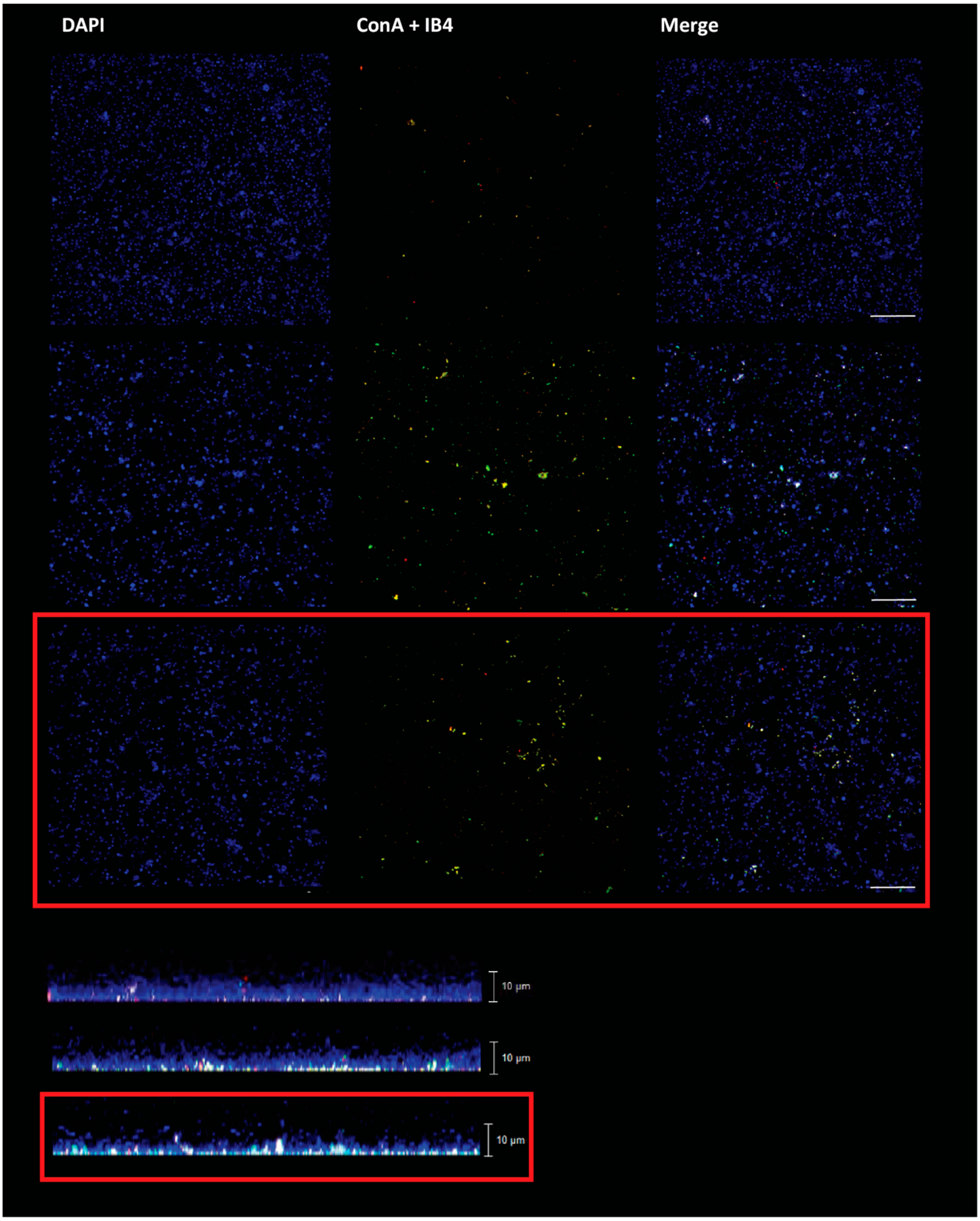

Figure S5. Replicates from biofilm CLM of *S. solfataricus* PolyP (-), at 0.5 mM Cu. The images used for the manuscript figures are indicated in red rectangles.

Table S1. Primers used in this work for qPCR.

Target	Sequence (5’ to 3’)
*arl*B Fw	TGCAGTAAACGAGATGGCTG
*arl*B Rv	TGACACAGAAAGCCAAATCCAC
*cop*A Fw	TGGTGTAAAAGATGCCGAAG
*cop*A Rv	TGGAGTAGTGGTCTCTGGATTG
SSO1101 Fw	GTTTTTCTAGATCTGCCTCC
SSO1101 Rv	GACTACTGATGATCTCTCCC
SSO3006 Fw	TCTATCCCTCATCCTTAGCG
SSO3006 Rv	CGTGGGATATCGAGAAGGG
16s Fw	GGTAGCAAGTAGACCCTATGC
16s Rv	GGTATCAGCAAAAGCGACAA
30s Fw	GGTAGCAAGTAGACCCTATGC
30s Rv	GGTATCAGCAAAAGCGACAA
